# Mutations in *BHD* and *TP53* genes, but not in *HNF1β* gene, in a large series of sporadic chromophobe renal cell carcinoma

**DOI:** 10.1038/sj.bjc.6603733

**Published:** 2007-04-17

**Authors:** S Gad, S H Lefèvre, S K Khoo, S Giraud, A Vieillefond, V Vasiliu, S Ferlicot, V Molinié, Y Denoux, N Thiounn, Y Chrétien, A Méjean, M Zerbib, G Benoît, J M Hervé, G Allègre, B Bressac-de Paillerets, B T Teh, S Richard

**Correction to**: *British Journal of Cancer* (2006) **96**, 336–340. doi:10.1038/6603492

Owing to a publishing error, part of the intended Supplementary Material for this paper was omitted at the time of publication. The correct supplementary tables were published; however, a further document was omitted. We are now happy to include this document as part of the Supplementary Material online – please visit the *British Journal of Cancer* website (http://www.nature.com/bjc).

